# Can Routine Anti‐Inflammatory Medications Reduce Population Dementia Risk?

**DOI:** 10.1002/alz.092406

**Published:** 2025-01-09

**Authors:** Connor Duncan Richardson

**Affiliations:** ^1^ Newcastle University, Newcastle Upon Tyne United Kingdom

## Abstract

**Background:**

Evidence from epidemiological and clinical studies of the effect of anti‐inflammatory medication on dementia risk has been mixed. Over the past two decades there has been recurring epidemiological evidence that the use of NSAID’s for chronic inflammatory conditions is associated with a lower incidence of dementia.

**Method:**

Cognitive Function and Ageing Study (CFAS) undertook baseline interviews in populations aged 65+ years in England and Wales (1989‐1994). Three areas (CFAS I) were selected for new sampling two decades later (2008‐2011) with same geographical boundaries, sampling and approach methods (CFAS II). At 2 years CFAS I interviewed 5,156 (76% response) with 5,288 interviewed in CFAS II (74% response). Data on medication use and dementia status was assessed at each wave.

**Result:**

Low dose aspirin (< 150 mg) use was associated by a reduction in dementia incidence rate (IR) in women from 60.99 (95% CI: 47.6, 78.2) per 1000 person‐years to 44.1 (95% CI: 2.0, 45.4) in 1993 and from 44.5 (33.7, 58.7) to 38.6 (27.2, 54.9) in 2013. No reduction in men was found.

**Conclusion:**

This study shows evidence that routine use of low‐dose aspirin reduces population risk of dementia in women. Mixed results in men show further analysis is needed to asses the effect over a longer follow‐up with a broader range of anti‐inflammatory medication.

